# Premises of social cognition: Newborns are sensitive to a direct *versus* a faraway gaze

**DOI:** 10.1038/s41598-020-66576-8

**Published:** 2020-06-17

**Authors:** Bahia Guellaï, Martine Hausberger, Adrien Chopin, Arlette Streri

**Affiliations:** 1 Bahia Guellaï, Laboratoire Ethologie, Cognition, Developpement, Universite Paris Nanterre, Nanterre, France; 2 Martine Hausberger, Universite de Rennes, CNRS, Laboratoire d’thologie animale et humaine, UMR 6552, Universite de Caen-Normandie, Caen, France; 3 Adrien Chopin, Institute of Vision, Aging in Vision and Action Lab, Sorbonne-Université, Paris, France; 4 Arlette Streri, INCC- UMR 8002, CNRS/Universite Rene Descartes, Centre Biomedical des Saints-Peres, 45, rue des Sts Pères, 75270, Paris, cedex 06 France

**Keywords:** Developmental biology, Social evolution

## Abstract

Previous studies evidenced that already from birth, newborns can perceive differences between a direct *versus* an averted gaze in faces both presented in static and interactive situations. It has been hypothesized that this early sensitivity would rely on modifications of the location of the iris (i.e. the darker part of the eye) in the sclera (i.e. the white part), or that it would be an outcome of newborns’ preference for configurations of faces with the eye region being more contrasted. One question still remains: What happens when the position of the iris is not modified in the sclera, but the look is ‘faraway’, that is when the gaze is toward the newborns’ face but above his or her own eyes? In the present study, we tested the influence of a direct *versus* a faraway gaze (i.e., two gazes that only differed slightly in the position of the iris on the vertical axis and not on the horizontal axis) on newborns’ face recognition. The procedure was identical to that used in previous studies: using a familiarization-test procedure, we familiarized two groups of newborns (N = 32) with videos of different talking faces that were presented with either a direct or a faraway gaze. Newborns were then tested with photographs of the face seen previously and of a new one. Results evidenced that newborns looked longer at the familiar face, but only in the direct gaze condition. These results suggest that, already from birth, infants can perceive slight differences of gazes when someone is addressing to them.

## Introduction

In many cultures, the eyes are often considered as the mirror of the soul because this region of the face is an important source of information in social interactions not only for human beings but also for many different species, especially mammals. Many examples from animals’ studies have evidenced that sensitivity to the direction of the gaze of others is deeply rooted in phylogeny. Jackdaws, for example, can detect the direction of gaze of both conspecifics and humans^1^. Domestic animals, such as dogs or horses are sensitive to the humans’ gaze direction: they obey less if the humans are not looking, have the eyes closed or have a distracted gaze^2–4^. Capuchin monkeys and infant chimpanzees react also differently according to humans’ gaze direction^5,6^. Thus, the sensitivity to conspecifics’ eyes is highly adaptive^7,8^ in some social species. There is a highly social significance of gaze direction^9^, and a special brain circuitry, involving amygdala^10,11^, has been evidenced. In human adults, this implication of a special circuitry has been confirmed, with an increase of arousal revealed through skin conductance^12^, heart rate^13^, BOLD responses in amygdala^14,15^, in response to a direct gaze compared to an averted one. Different studies agree that eye contact is a major non-verbal channel for communication^16^.

Indeed, in humans, direct gaze can constitute an important social cue engaging its target in a social interaction^16^. As evidenced in previous work, different social functions of the human eye gaze can be identified, including following of someone’s gaze to significant objects^17,18^, regulating turn-taking in conversation^19,20^, expressing intimacy^21,22^, and inferring mental states^23^. The direction of gaze can also influence social knowledge about others^24^. Results of a behavioral study showed that perceived eye gaze can modulate face recognition both at the encoding and retrieval levels, with better performances when facing someone with a direct gaze *versus* a gaze directed aside, both in adults and children^25^. The same finding has been observed in early infancy^26^: 4-months-old infants recognized the previously seen photograph of a woman’s face, as evidenced by a novelty preference, only if the face was first seen with a direct rather than with a gaze directed aside or with closed eyes. By 4-months of age, enhanced neural processing of direct gaze has also been observed^27^.

As direct gaze seems to play an important role for the development of social skills, the question of the timing and mechanisms involved in the emergence of gaze direction detection is of primary importance. Different studies have evidenced that infants, from birth, are already sensitive to the gaze of others, and they prefer to look at the photograph of a face with the eyes open *versus* closed^27^. They also prefer looking at a photograph of a face with direct *versus* averted gazes^27^. In a series of experiments, Farroni and colleagues^26–28^ evidenced that newborn infants were able to differentiate (and preferred) a direct from an averted gaze, whether on actual photographs or schematic faces. Nonetheless, the underlying mechanisms driving this early sensitivity remain unclear, as Farroni and colleagues mentioned^29^: “Experiments need to be done to determine the basis of this preference in newborns”. While some authors such as Baron-Cohen^23^ proposed an ancient evolutionary mechanism, shared with many animal species (i.e., the ‘EDD’, Eye Direction Discrimination) as an innate mechanism to explain this phenomenon, Farroni and colleagues^27,29^ proposed that this ability would just be an outcome of the newborns’ preference for the configurational organization of faces. Indeed, some authors^30^ have hypothesized that the preference for faces is based on a primitive representation of high contrast elements (i.e., location of eyes and mouth), and Farroni and colleagues^31^ suggested that direct gaze just fits better with the spatial organization of elements in this general template than a gaze averted to the right or the left (i.e., in a direct gaze the eye balls are symmetrical but not in a gaze directed aside). Also based on contrast detection, another possibility is that the symmetrical (i.e., direct gaze) *versus* the asymmetrical (i.e., averted sideways gaze) gazes induce important contrast differences in terms of ratio of exposed sclera^32,33^. In Farroni and colleagues’ study^29^, removing the sclera did not prevent newborns to follow gaze cues, suggesting that they relied upon the higher contrast of the eyes, rather than eye motion *per se*. This does not however fully explain the preferences, beyond gaze following, for static direct gazes at birth.

There are two aspects to be considered to understand the early sensitivity for a direct gaze: (1) As a result of evolutionary history, it is possible that in the first hours of postpartum experience, newborns are ‘equipped’ with the premises of the detection of attention directed to them (i.e. gaze directed to them or not), and thus may rely upon gaze direction *per se* rather than just symmetry or the overall face representation. One way to test this possibility is to present a ‘faraway’ gaze that does not rely upon changes in contrast or symmetry compared to a direct gaze. In fact, different animal species have been shown to discriminate an ‘eye-ceiling’, or ‘eye above target’ from a direct gaze in humans (i.e. human looking above the eye of the animal)^34,35^; (2) In daily interactions, faces are never seen static: they talk, laugh, and move. In these more complex situations, other cues such as speech seem to modulate attention to the eye region which may influence face processing already from birth as evidenced in various studies^36–39^. In these studies, authors used the same procedure closed to ecological settings: newborns were first familiarized with videos of a woman’s face in different conditions (i.e., talking or silent, talking while looking at the newborn or while looking aside, with speech being low-pass filtered or not…). Then, in a recognition test phase, photographs of the previously seen woman and of a new one were presented in an alternated manner. Moreover, authors voluntarily used highly contrasted faces to test whether the sensitivity to different cues such as gaze direction may be important enough to overcome the discrimination of other more contrasted face characteristics. Results of these studies evidenced, for example, that direct gaze alone (i.e., without verbal interaction) is not a sufficient cue in guiding newborns’ recognition of a previously seen face: Newborns preferred looking at a woman who previously looked at them and interacted with them verbally, but not at a woman who looked at them without speech sound^36^. Another study evidenced that in these interactive situations, it is both speech and direct gaze that are important for newborns’ recognition of a face^37^. That is, newborns were sensitive to both socio-communicative cues, speech and direct gaze in these ecological situations. One possibility to explain these results is that newborns are already sensitive to social situations in which someone is addressing to them with a mutual gaze rather than with a gaze not directed to them.

In the present study, we propose to use the same procedure already used in earlier studies^36–39^, to test the following possibilities:

1. Newborns do have a sensitivity for a mutual gaze *per se*, as a result of both human evolutionary history and maybe the first hours of postpartum experience, and not just because of contrast sensitivity. Therefore, we used this ‘eye above target’ type of stimulus as an alternative to the commonly used ‘gaze directed aside’ to test whether human newborns do prefer mutual gaze, even when both the face template and the eyes’ contrast symmetry are kept. The ‘eye above target’ gaze is typical when someone is facing another person with the eyes gazing slightly above the other’s eyes, as if looking faraway. Contrarily to the averted gaze, it displays a slight vertical and not horizontal offset position of the iris. Therefore, it provides the same contrast as a direct gaze (i.e., same amount of white on both sides of the eyeball) but is not mutual. Besides their contrast similarities, the two gaze directions are interesting to test as they give different information about the speaker’s communicative intentions and would be therefore powerful social cues. Indeed, a direct gaze is conductive to an interaction whereas a (here called) faraway one involves no eye contact and therefore would disrupt the interaction. To our knowledge, the comparison of the influence these two types of gaze on face recognition has never been tested in humans.
2. It is possible that the early sensitivity (and preference) for direct *versus* averted gazes is enhanced by ecologically valid situations of social interactions and is involved in individual face processing. Therefore, we used a familiarization-test procedure with audiovisual presentations of faces. After a familiarization phase with the video of a woman talking with either a direct or a faraway gaze, the previously seen face and a novel one were presented in a test phase. If contrast sensitivity (e.g. symmetry of sclera contrast, overall face template) is the most important cue, newborns would recognize the familiar face in either situations, with a direct and with a faraway gaze. If newborns are already sensitive to direct gaze as being socially engaging, following previous findings^36,37^, we expect newborns to look more at someone who previously talked to them with a direct rather than with a faraway gaze. Moreover, we voluntarily used highly contrasted faces to test whether the sensitivity to gaze direction may be important enough to overcome the discrimination of other more contrasted face characteristics such as hair color. This choice was based upon earlier studies that showed that some visual social cues were processed preferentially to global face characteristics at birth^36–39^.

## Methods

We adapted the procedure used in previous studies with interactive faces presented in videos^37^ to test newborns’ sensitivity to a mutual *versus* a faraway gaze. In a familiarization phase, newborns saw an unfamiliar woman talking to them either with a direct or a faraway gaze. In a recognition test phase, newborns were exposed to the same woman and to a new one. The durations of looking times toward each face were recorded.

### Participants

The final sample included 32 healthy full-term newborns (18 boys) from a maternity hospital in France. All infants had an APGAR score of 10 after five minutes. The mean age was 47 hours (*SE* = 13 h, range: 26h-72h). Thirteen additional infants were excluded from the analysis due to sleepiness (6), fussiness (2), cries (4) or technical problem (1). In these cases, newborns did not complete the procedure and rejection criteria was decided by the two experimenters without looking at the data. All newborns were tested when they were awake and in an alert state. The protocol was carried out in accordance with the ethical standards of the Declaration of Helsinki (BMJ 1991; 302:1194) and approved by the Ethic Committee of the Department of Psychology of the University Paris Nanterre (Ethic Committee Number 2019-7). Written informed consent was obtained from the parents prior to the testing.

### Apparatus

As described in our previous work^37^, newborns were observed in a quiet room accompanied by one or both parents. Before testing, we systematically ensured that parents and medical staff gave their agreement. Each newborn was positioned in a semi-upright position (30°) in an adapted rigid seat placed on a table facing a 19-inch DELL color monitor, 35 cm away from the infant’s eyes. Two speakers were placed on each side of the monitor. One experimenter (Experimenter 1) stood behind the newborn during the whole session to monitor for potential signs of discomfort. A video camera was directed at the newborn, recorded the whole experiment (the temporal resolution was 25 images/s), and displayed the images on two video monitors. One monitor allowed a second experimenter (Experimenter 2) to code the duration of looking. The other allowed the parents to see their baby. The parents sat behind and far from the baby, so that the infant could not see them. Parents were instructed to not intervene (speak or come near their baby) during the duration of the experiment.

### Stimuli

**Figure 1:**
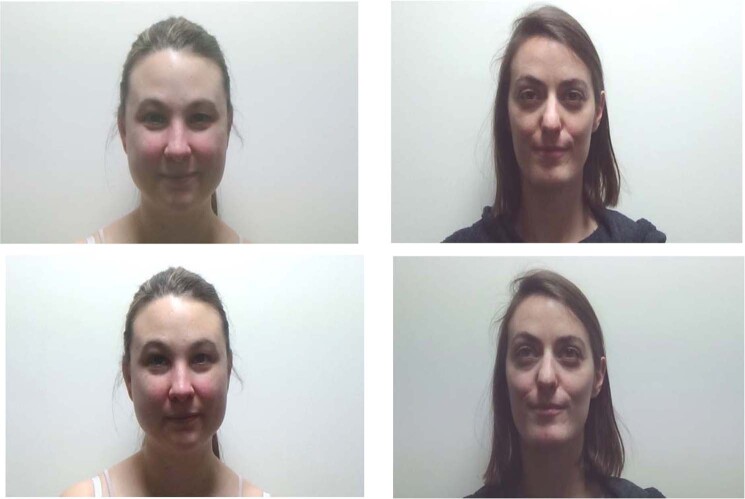
Examples of the stimuli presented: women with a direct gaze (on the top) *versus* a faraway gaze (on the bottom).

For the familiarization phase, four different color video clips of two women were recorded. Informed written consents were obtained from the two women for video recordings and to publish their information/images in an online open-access publication. These videos were recorded under the same lighting conditions (mean: 16 cd/m2) with the same black background in a soundproof room. Since the aim of the study was to test whether recognition of a face can be influenced by the type of gaze associated with it, we chose two highly contrasted women’ faces: One with short dark brown hair (Woman 1) and the other one with long lighter brown hair (Woman 2). Both had brown iris. This enhancement of hair contrast enable to ensure that the potential newborn’s failure to recognize one face over the other could not be due to a mere lack of perceptual discrimination of the overall facial features as half of participants were presented with Woman 1 and the other half with Woman 2 during the familiarization phase. Thus, in the recognition test phase, Woman 1 was familiar, and Woman 2 was unfamiliar for half of the newborns whereas Woman 1 was unfamiliar, and Woman 2 was familiar for the other half. The two women were native French speakers. They both had previously learned the same text and while video recorded, each woman addressed to the camera in an Infant-Directed Speech (IDS) style while looking directly to the camera (i.e., mutual gaze condition) or looking above the camera (i.e., faraway gaze condition) (see Fig. 1). Following methodology presented in Mayhew and Gomez^40^, we calculated the amount of white sclera for all stimuli. In the mutual gaze condition, amount of white sclera represented 15% on the left and 15% on the right parts of the eyeball and in the faraway gaze condition 15% on the left and 15% on the right. The deviation of the center of the eyeball calculated on an abscissa axis was 0 for the mutual gaze condition and +2 for the distracted gaze condition.

Similar to stimuli described in our previous work^37^, each of the four videos lasted for 75 seconds. Sound intensities at the speakers in the testing room were identical for all stimuli (65 dB). For the recognition test phase, the last frame of each familiarization video clip was presented without motion (i.e., static), independently of the position of the head. All facial images in the familiarization and in the recognition test phases were presented at life size. Each image subtended a visual angle of 41 × 36° and the external contour of one eye was approximately 3 × 6°.

### Procedure

Thirty-two newborns participated to our experiment. Half of the newborns (n = 16) was presented with faces displaying a direct gaze whereas the other half was presented with faces displaying a faraway gaze. The experiment began as the infant was seated. During a familiarization phase, newborns were presented with either Woman 1 or Woman 2 talking to them. for 75 seconds. Immediately after the familiarization phase, the recognition test phase began. Newborn saw the photograph of the familiar face (F), that is the woman presented during the familiarization phase, and the photograph of a new one (N) in an alternated manner. Half of the newborns saw the two faces in the FNFN order and the other half in the NFNF order. A computer program randomly determined which of the four conditions was presented to each of the participants: Familiarization (Woman 1 or Woman 2) and Test (FNFN or NFNF).

During the familiarization phase (i.e., lasting for 75 seconds), an experimenter, unaware of the face presented, pressed and held a key button on a computer keyboard when the infant looked at the screen and released it when the infant looked away. A computer program recorded the accumulated looking times. During the test phase, the experimenter proceeded in the same way, but when newborns looked away from the screen for more than two seconds, the computer program automatically switched to the next stimulus. A switch also occurred after the newborns had looked at the face for 60 seconds continuously (i.e., maximum length of each video in the test phase). The computer program also required a minimum of 2 seconds looking time at the screen. Looking times were verified *a posteriori* from the video recordings by another experimenter, blind to the experimental conditions. Inter-observer reliability was assessed by the two Experimenters blind to the conditions while coding (Pearson’s *r* = 0.89, *p* < 0.01).

## Results

### Statistical analysis

The looking times toward the faces were recorded for each infant as the dependent measure. We analyzed the data collected using the MatLab program. We first checked for the normal distribution of the looking times in the faraway gaze and the direct gaze groups during the familiarization phase: Kolmogorov-Smirnov test (direct gaze): hyp H0, KS = 0.176, p = 0.6433412, and Kolmogorov-Smirnov test (faraway gaze): hyp H0, KS = 0.194, p = 0.5247387. Results showed that distributions did not differ significantly from normal through KS tests in the familiarization phase.

We also checked for the normal distribution of the data during the test phase. In the faraway condition, for each block of presentation, with Kolmogorov-Smirnov tests: Woman 1 (first block): hyp H0, KS = 0.168, p = 0.6969990; Woman 2 (first block): hyp H0, KS = 0.209, p = 0.4308260; Woman 1 (second block): hyp H0, KS = 0.185, p = 0.5781236; Woman 2 (second block): hyp H0, KS = 0.258, p = 0.2005872. In the Faraway gaze condition, for each block of presentation: Woman 1 (first block): hyp H0, KS = 0.262, p = 0.1848129; Woman 2 (first block): hyp H0, KS = 0.233, p = 0.3027491; Woman 1 (second block): hyp H0, KS = 0.212, p = 0.4133000; Woman 2 (second block): hyp H0, KS = 0.220, p = 0.3667597. So, none of the face presentation looking times at Test followed distributions significantly different from a normal one.

### Familiarization phase

**Figure 2:**
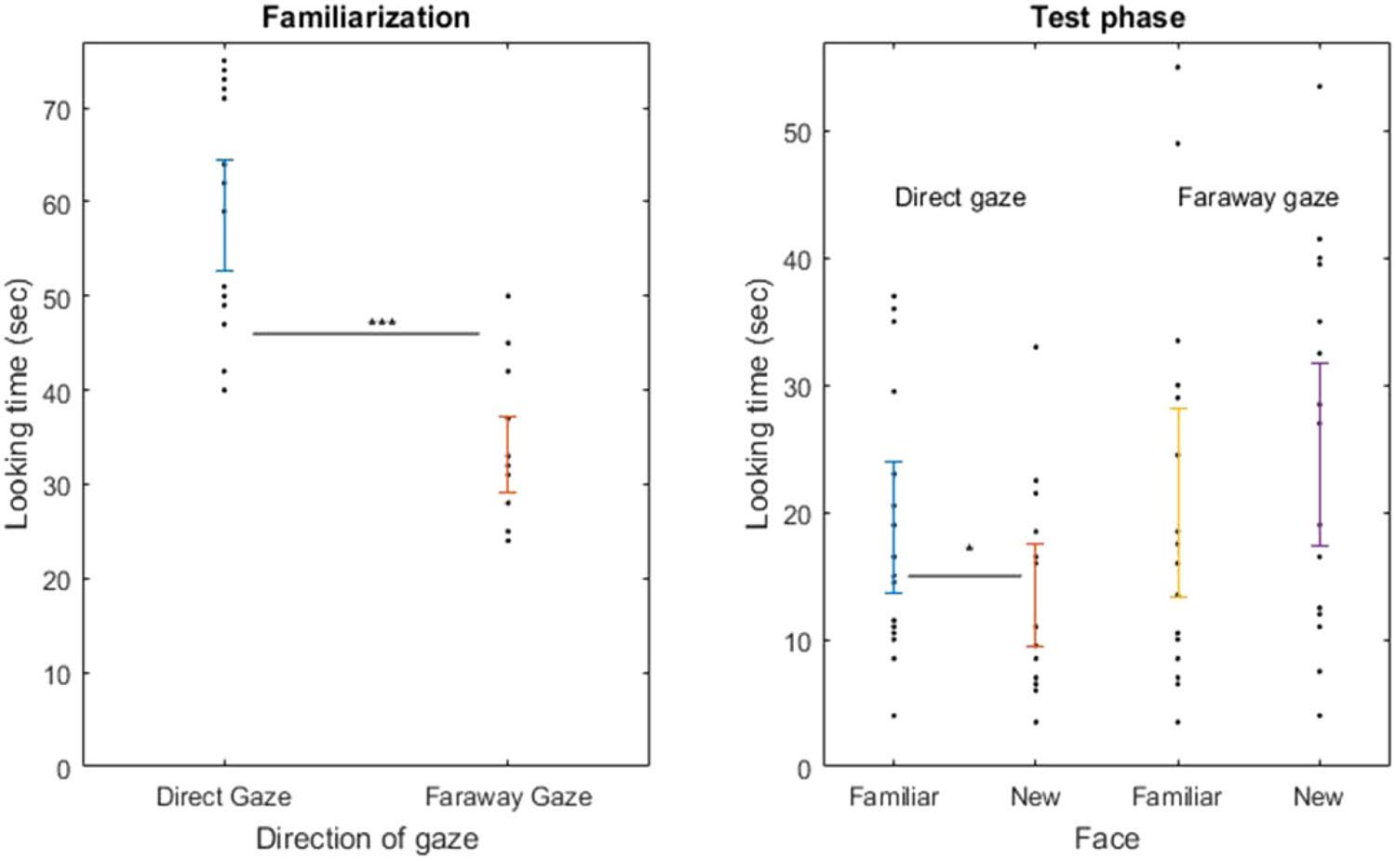
Looking times of participants in front of the faces presented during the familiarization and test phases. ***p < 0.001, *p < 0.05.

We tested whether newborns’ attention was affected by the type of gaze during the familiarization phase by comparing the duration of newborns’ looking behaviors across this phase. Looking times of newborns watching a talking face with a direct gaze were longer than those of newborns watching talking faces in the faraway gaze condition (direct gaze: 58.6 sec., SE = 11.94; faraway gaze: 33.13 sec., SE = 8.27; independent sample t-test: *t*(30) = 7,01, p = 0.0000001, Cohen’s d = 2.481), whatever the face was (Woman 1 or Woman 2) (see Fig. 2). Thus, although half (16) of the 32 newborns were familiarized with Woman 1 and the other half with Woman 2, there was no significant difference in mean looking times according to the two faces (Woman 1: 45 sec., SE = 4.03; Woman 2: 46.75 sec., SE = 4.29; independent sample t-test: *t*(30) = −0.29, p = 0.76, Cohen’s d = 0.42). These results show that newborns are sensitive to differences of orientation of the iris in talking faces, and that someone talking to them with a direct gaze triggers longer familiarization times than someone with a faraway gaze.

### Recognition test phase

During the recognition test phase, mean looking times to the familiar and to the new faces were analyzed in both the direct and the faraway gaze conditions (see Fig. 2). Twelve out of the sixteen newborns in the direct gaze condition looked longer at the familiar face (familiar face: 37.69 sec., SE = 5.24; novel face: 26.87 sec.). Thus, most of them recognized the face seen during the familiarization phase. In the faraway gaze condition, only 6 out of the 16 newborns looked longer at the familiar face (familiar face: 41.56 sec., SE = 7.58; novel face: 49.06 sec., SE = 7.33).

We checked if the looking times were different between first and second face presentations. In the direct gaze group, first and second presentation of Woman 1: *t*(30) = 0.878, p = 0.387, Cohen’s d = 0.310; first and second presentations of Woman 2: *t*(30) = −0.180, p = 0.859, Cohen’s d = −0.064. In the faraway gaze group: first and second presentation of Woman 1: *t*(30) = 1.293, p = 0.206, Cohen’s d = 0.457; First and second presentation of Woman 2: *t*(30) = 1.471, p = 0.152, Cohen’s d = 0.52. As they were not different, we averaged them together.

A mixed design ANOVA with direction of gaze as a between-subject factor (direct *versus* faraway gaze) and familiarity of face (familiar *versus* new faces) as a within-subject factor was performed on looking times: direct gaze *vs*. faraway gaze: *F*(1,30) = 2.838, p = 0.102, *η²*= 0.086; familiar *vs*. new faces: *F*(1,30) = 0.153, p = 0.699, *η﻿²* = 0.005; interaction: *F*(1,30) = 4.663, p = 0.039, *η﻿²* = 0.135. The ANOVA evidenced a crossed interaction for the familiarity of face factor, so that one cannot conclude to an absence of difference on the levels on that factor. For the direct gaze group, familiar faces are gazed significantly longer than new faces but not in the faraway gaze group.

However, to confirm that there was no difference between the familiar and new faces in the faraway gaze group, we did additional post-hoc paired sample t-tests on the comparisons: familiar-new in the direct gaze group t-test: *t*(15) = 2.188, p = 0.0449352, Cohen’s d = 0.547; familiar-new in the faraway gaze group: *t*(15) = −1.088, p = 0.2936360, Cohen’s d = −0.272. In the direct gaze group, neonates looked at the familiar face significantly longer than at the new face but not in the faraway gaze group. There was no difference of looking times according to the individual, Woman 1 or Woman 2, in both conditions (t-tests, direct gaze: *t*(30) = 0.06, p = 0.95, Cohen’s d = 0.72; faraway gaze: *t*(30) = −0.44, p = 0.65, Cohen’s d = 1.44). No other effect or interaction was significant.

## Discussion

The results of the present study showed that: 1/ during the familiarization phase, newborns watching persons talking to them in the direct gaze condition looked longer at those faces than newborns presented with the same individuals in the faraway gaze condition, suggesting that direct gaze enhances newborns’ attention to the speaker, with no differences according to the physical characteristics of the individuals; and 2/during the recognition test phase, only the newborns in the direct gaze condition looked longer at the face seen during the familiarization phase whereas there was no difference of looking times between the familiar and new faces for those in the faraway condition, no matter the individual, Woman 1 or Woman 2.

These results confirmed our hypotheses that: (1) newborns are indeed able to discriminate a faraway gaze from a direct one and thus do not necessarily rely upon mere contrast to assess gaze direction, and (2) this ability is enhanced by an ecologically relevant social situation (i.e. talking face) which influence newborns’ face processing. Indeed, during the familiarization phase, newborns who were presented with a woman talking to them with a direct gaze looked longer at her than those who were presented with the same woman with a faraway gaze. Moreover, in the recognition test phase, only the newborns in the direct gaze condition looked longer at the familiar face compared to a novel one.

Previous research using photographs of faces evidenced the importance of a direct gaze compared to a gaze directed aside^27,29^, or a face with closed eyes^41^ on face processing at birth. Other studies, using audiovisual dynamic faces, evidenced that newborns can recognize a woman who previously talked to them with a direct *versus* an averted gaze^37^, which was a gaze directed aside. A possible interpretation was that in these experiments, newborns’ sensitivity relied mainly on contrast properties due to the iris position and not on the social aspect of interaction. The present study, in which both gazes presented the same contrast distribution in the face template and contrast symmetry, demonstrates that the anatomic explanation of eyes and its contrast/directions^26^ is not the sole possible explanation. These results also suggest that visual abilities of newborns may have been underestimated, as suggested by some authors^42^: Visual acuity and distance perception have not been revisited since Fantz^43^ and Teller^44^ earlier studies. For example, further studies have shown, through forced choice preferential looking, that newborns can see up to 1 m, thus far more than the 18–25 cms suggested by Fantz’ studies^42^. Even though the central zone of the retina involved in accurate perception is not fully developed at birth^45^, previous studies have shown that newborns are able to process subtle visual cues, such as the congruence between lip movements and speech composition^46^. In fact, earlier perceptual studies used non-ecological stimuli whereas newborns appear to be attentive to consistent and living stimulation^38^. As social attention may modify perceptual processing in other species and at later stages of development^47,48^, it would be very interesting to develop further studies on newborns. The results obtained in previous studies^36–39^ and the present one converge to show that newborns seem to be sensitive to someone who is engaging them in a face-to-face interaction rather than, as shown here, someone who is talking and looking toward them but not at them.

This is probably why ecologically relevant social cues are so important already from birth. Thus, results of the present experiment (i.e., newborns looked longer at faces seen previously with a mutual rather than with a faraway gaze), extends those observed in previous studies: Newborns looked longer at photographs of familiar faces only when those persons were previously seen talking with a direct gaze^36,37^. It is possible that longer looking times at the face in the direct gaze condition during the familiarization phase would have triggered more attention to the face and therefore improved the encoding of the face such as it has been shown in experiments with adults^41^ and infants^31^. This result is also in line with other studies showing that newborns could identify emotions in the photograph of a face only when they had been previously habituated to this face with a direct *versus* an averted gaze^49^, and that 4-month-old infants preferred to look at faces with direct eye contact rather than averted eye gaze^26^. Overall, previous results evidenced the importance of direct gaze in face processing already from birth and its possible implication in the development of early social skills.

Taking together, these results are in line with both Baron-Cohen and colleagues’ theory that human eyes play a predominant role in the early face processing system *via* the existence of an ‘Eye Direction Detector’ (EDD)^49^, and Farroni and colleagues’ position^27^ that newborns are “equipped” with basic skills that will support the development of social skills and especially the ability to detect others’ attentional skills, a capacity demonstrated in an array of animal species, and further enhanced through social experiences. For Baron-Cohen and his colleagues, the system first detects the presence of eyes and then codes their direction. Our previous experiments^36,37^ provided evidence that the direct gaze without speech does not allow newborns to recognize the familiar face. Here, we therefore used talking faces and the results obtained are probably due to both the presence of speech and direct gaze that enhance face recognition at birth. At that stage, the exact significance of direct gaze for the newborns is not fully understood, and one can only speculate that a distracted gaze (whether as a faraway gaze or a gaze directed aside) may be perceived as if the speaker does not address to the infant directly, but is talking to someone else for example, and therefore as breaking the dyadic interaction may affect face processing. Further studies could use different speech registers (i.e., an Infant-Directed Speech or an Adult-Directed Speech) to go further in these interpretations, and to test if newborns are already sensitive to an addressed speech.

In conclusion, the present study demonstrates further the early ability of human newborns to discriminate whether adults address to them with a directed or a distracted gaze, but here it has been evidenced that this is not merely due to the contrasts perceived in the eye region. It shows how important the social context may be to trigger and reveal such an ability and how this early sensitivity to direct gaze combined with live social experiences may help transform an early ability, issued from a long evolutionary history, into proper complex social skills, such as the later ability to discriminate the interlocutors’ attentional state^50^. Because social interactions and early perceptual abilities mutually influence the development of social cognition, these results may be of importance for caretakers and parents, encouraging them to actively interact with newborns without being distracted by other stimuli.
